# From Aid to Impact: The Cost-Effectiveness of Global Health Aid in Sub-Saharan Africa and the Evolving Role of Microinsurance

**DOI:** 10.3390/healthcare13141716

**Published:** 2025-07-16

**Authors:** Symeon Sidiropoulos, Alkinoos Emmanouil-Kalos, Michail Chouzouris, Panos Xenos, Athanassios Vozikis

**Affiliations:** 1 Department of Public and One Health, University of Thessaly, 43100 Karditsa, Greece; 2 Hellenic Association of Political Scientists, 10673 Athens, Greece; a.emmanouil-kalos@unipi.gr; 3 Laboratory of Health Economics and Management, University of Piraeus, 18534 Piraeus, Greece; avozik@unipi.gr; 4 Department of Economics, University of Piraeus, 18534 Piraeus, Greece; 5 Department of Statistics and Insurance Science, University of Piraeus, 18534 Piraeus, Greece; mchouzouris@unipi.gr (M.C.); pxenos@unipi.gr (P.X.)

**Keywords:** Development Assistance for Health, communicable diseases, health systems strengthening, HIV, malaria, tuberculosis

## Abstract

**Background**: Development Assistance for Health (DAH) plays a vital role in health financing across Sub-Saharan Africa, particularly in tackling communicable diseases such as HIV/AIDS, malaria, and tuberculosis. Despite its importance, the efficiency and equity of DAH allocation remain contested. **Objectives:** The study aims to evaluate the cost-effectiveness of DAH in Sub-Saharan Africa from 1995 to 2018, as well as to explore differences in efficiency across diseases and country contexts. **Methods**: Data were drawn from the Institute for Health Metrics and Evaluation and applied Generalized Cost-Effectiveness Analysis in conjunction with the Gross Domestic Product-based thresholds. Averted Disability-Adjusted Life Years were analyzed across countries and diseases, and countries were categorized by the Human Development Index (HDI) level to assess differential DAH performance. **Results**: DAH cost-effectiveness showed similar patterns across HDI groups, with roughly equal proportions of cost-effective and dominated outcomes in both low- and middle-HDI countries. Thirteen countries were identified as very cost-effective, nine as cost-effective, and two as non-cost-effective. Twenty-one countries were dominated, reflecting persistent inefficiencies in aid impact that transcends the various levels of development. **Conclusions**: Tailoring DAH allocation to specific disease burdens and development levels enhances its impact. The study underscores the need for targeted investment and a strategic shift toward integrated health system strengthening. Additionally, microinsurance is highlighted as a key mechanism for improving healthcare access and financial protection in low-income settings.

## 1. Introduction

Development Assistance for Health (DAH) is a fundamental tool of international aid primarily provided by developed economies, employing strict mechanisms of transparency and evaluation over the past two decades [1,2]. DAH fosters a more favorable environment for addressing infectious diseases, epidemics, and pandemic crises in general [3]. According to data from the Institute for Health Metrics and Evaluation (IHME) [4], global DAH accounted for 0.046% of the global Gross Domestic Product (GDP) in 2018, corresponding to $39 billion. In the case of the Sub-Saharan Africa region, however, this percentage was significantly higher, amounting to 0.65% of the region’s GDP, equivalent to $10 billion. Moreover, in the same year, DAH represented 0.2% of global health expenditures, while it accounted for 13.17% of total health expenditures in Sub-Saharan Africa.

These figures underscore that, while DAH may appear relatively insignificant at a global scale, it serves as a critical financing mechanism for health systems in the region under study (and in other developing regions), as evidenced by its substantial share of total health expenditures there. Within the region, however, the economic capacities of individual countries vary considerably, as does their reliance on DAH. Based on the World Bank classification, this variation reflects the presence of low-, lower-middle-, and upper-middle-income countries, which influences the overall percentage of GDP and health expenditures attributed to DAH. It is worth noting that DAH has seen remarkable growth, increasing from $9 billion in 1990 to $67 billion in 2021. This sharp rise in DAH funding has inevitably drawn continuous scrutiny regarding the tools and mechanisms employed, alongside a persistent demand for improvement to enhance its effectiveness [5].

As Radelet [6] observed, a substantial proportion of aid has historically been delivered through partnerships involving multiple stakeholders. Enhanced provision is primarily achieved through a “circular” financial flow, wherein fragmented contributions from various sources are consolidated. Key actors in this interactive process of DAH include the World Bank, the International Monetary Fund, the African, Asian, and Inter-American Development Banks, as well as specialized United Nations agencies and programs, such as the United Nations Development Program.

Health crisis management is closely linked to controlling communicable diseases. Unsurprisingly, DAH is primarily directed toward combating communicable diseases, including sexually transmitted infections and respiratory illnesses, among others. In 2018, HIV/AIDS, tuberculosis, and malaria accounted for nearly one-quarter of DAH, while, together with other infectious diseases, they comprised more than 50% of total funding. Notably, a significant portion of DAH consistently supports maternal and child health initiatives.

In contrast, Non-Communicable Diseases (NCDs) received only 1.62% of the total funding [4]. Despite this limited allocation, Ruby et al. [7] highlighted the growing challenge posed by NCDs even in low-income countries. In high-income countries, conditions such as ischemic heart disease, cardiovascular disorders, and cancer represent leading causes of death, driven primarily by dietary habits, stress, and other lifestyle factors. In Sub-Saharan Africa, specifically, NCDs are projected to become the leading cause of mortality by 2030 [8]. This underscores the pressing need for greater investment in addressing these emerging health burdens in low-resource settings.

Focusing on communicable diseases, which, as noted earlier, represent the primary target of DAH, and specifically on HIV, tuberculosis, and malaria—the diseases under study—it becomes evident that a significant portion of aid is allocated to interventions aimed at disease management and treatment. More specifically, funding flows directed toward HIV treatment account for 33.3% of the total funds allocated to addressing the disease. Similarly, treatment-focused funding constitutes 17.1% of malaria-related DAH and 13.5% of tuberculosis-related DAH [4]. This stands in stark contrast to the management of these diseases, particularly HIV/AIDS, which are managed in the developed world, where they often present as chronic conditions rather than acute threats. This disparity highlights the multifaceted contributors to the observed outcomes, including the overall state of the health system, the effectiveness of preventive measures, public health education, treatment availability, and adherence to prescribed therapies. These factors collectively shape the negative impact of these infections and are critical to addressing their burden in the region.

Non-adherence to prescribed treatments, which is documented at high rates in Sub-Saharan Africa, contributes significantly to the development of drug-resistant viral strains [9,10]. According to Heestermans et al. [9], patient “refusal” to follow scheduled treatments is a frequent phenomenon in the region. This non-compliance is often linked to systemic barriers, particularly in impoverished areas, where patients are required to collect their medications from service centers (pharmacies or clinics) located in remote and hard-to-reach areas [9]. According to the same study, another critical factor contributing to non-adherence is the lack of personalized care. After enduring lengthy and arduous journeys, patients often face overcrowded service centers where treatments are dispensed en masse. This impersonal approach, coupled with poor service quality, has been identified as a major source of patient dissatisfaction, further exacerbating non-adherence [9]. The poor quality of care can also be attributed to healthcare worker burnout, as highlighted by the same study. This is further substantiated by data from the World Health Organization [11], which reports striking disparities in healthcare workforce availability. In Sub-Saharan Africa, the physician-to-population ratio is 1 per 3623 individuals, and the nurse-to-population ratio is 1 per 972. By contrast, in developed regions such as Europe, these ratios stand at 1 per 232 and 1 per 129, respectively. These stark differences underscore the systemic challenges contributing to inadequate patient care and subsequent non-compliance in the region.

Given the central role that DAH plays in financing health systems across Sub-Saharan Africa—and the growing demand for accountability and impact—this study aims to evaluate the cost-effectiveness of DAH in relation to the region’s three most heavily funded communicable diseases: HIV/AIDS, malaria, and tuberculosis. The research focuses on two key dimensions of analysis: first, identifying which of the three diseases demonstrates the most efficient use of DAH as measured by averted Disability-Adjusted Life Years (DALYs); and second, determining which countries in Sub-Saharan Africa have absorbed DAH most effectively in terms of health outcomes. In addition, the study explores how cost-effectiveness varies according to the Human Development Index (HDI) classification of recipient countries. These questions are addressed through a multidimensional approach that combines statistical analysis, cost-effectiveness metrics based on GDP per capita, and visual mapping techniques. Finally, the presented results lay the groundwork for a discussion on how insurance could contribute to improving health. In this manner, a brief discussion is presented on plausible solutions and examples of implemented policies using microinsurance, an essential tool for enhancing financial inclusion and reducing vulnerability among low-income populations.

The paper is structured as follows: the next section describes the methodology and data sources used in the analysis, followed by a detailed presentation of results, a discussion placing the findings within the broader literature on DAH, and final thoughts addressing with implications for future policy and research.

## 2. Materials and Methods

A consolidated dataset was constructed by integrating data on global health expenditures (1995–2019) from the Global Burden of Disease Collaborative Network [12], data on the worldwide burden of disease (1990–2019), and data on DAH (1990–2019) from IHME [4,13]. These datasets were combined due to their complementarity in providing the necessary data for the analysis and their shared coding structure (as they originate from the same institution), facilitating secure data management and processing. The research involved a series of methodological steps, including quality control of the raw data, selection and formatting of the required variables, integration of the datasets, and the creation of new calculated variables to depict cumulative values and ensure comparability across the study period (1995–2018). Year-on-year reductions in DALYs are used as a proxy for potential averted burden. DALYs are used as published by the Global Burden of Disease study, which incorporates standardized global disability weights.

The analysis adopts a donor perspective, focusing on the efficiency of DAH in generating health outcomes across recipient countries. To assess whether, and to what extent, DAH contributes to addressing the three diseases in question (HIV/AIDS, malaria, tuberculosis)—more specifically, in reducing DALYs—and potentially under what conditions this occurs, we first categorize the countries of Sub-Saharan Africa based on their HDI. The HDI provides a composite measure of average achievement across three fundamental dimensions of human development: longevity and healthy life, access to education, and a decent standard of living [14]. It is calculated as the geometric mean of normalized indices for these three dimensions. Based on the HDI categorization, the Sub-Saharan countries are divided into three groups: low HDI (<0.55), middle HDI (0.56–0.70), and high HDI (>0.71).

In addition to total DAH, the analysis incorporates other important country-level factors to control for differences in health system capacity and population health status. These include average health spending per capita, physician density (measured as physicians per 1000 people), and immunization coverage of DPT (diphtheria, pertussis, and tetanus) among children aged 12–23 months. These variables help contextualize the effectiveness of DAH in reducing DALYs by accounting for variations in healthcare infrastructure and preventive health measures.

The following regression model was used in an exploratory capacity to examine the associations between DAH and averted DALYs across HDI groups, controlling for key health system and population factors:

*Aggregate Averted DALYsi* = *β*0*i* + *β*1*Physiciansi* + *β*2*DPTi* + *ε*

where *i* = reference disease (1, 2, and 3, correspondingly), *Physicians* = Physicians per 1000 people, and *DPT* = immunization coverage (% DPT vaccinated children)

Additionally, to evaluate the cost-effectiveness ratio—specifically, DAH-to-DALYs averted—GDP per capita data (constant 2018 USD) were obtained from the World Development Indicators database of the World Bank. This supplementary dataset enabled the calculation of the cost-effectiveness metrics critical to the analysis. The Generalized Cost-Effectiveness Analysis (GCEA) was selected as best suited for our study, as it offers the possibility to assess the effectiveness of an intervention compared to what would have resulted if an intervention had not been realized. As Rubinstein et al. [15] mentioned, GCEA “…is a methodology designed by the WHO to evaluate the current and potential coverage of health interventions in order to improve allocative efficiency and to facilitate policymakers’ ability to make informed decisions about health resource allocation”. Furthermore, it enables us to compare the effectiveness of interventions in different countries [16], which comprises one of the main subjects for analysis of this study. The effectiveness of the assistance provided to the reference countries is performed by considering the cost-effectiveness ratio of DAH-to-DALYs averted in conjunction with the average GDP per capita for each country from 1995 to 2018. For the evaluation, the World Health Organization’s [17] proposed threshold values were applied, which specify the following: (a) if DAH/DALYs averted < 1× GDP per capita, then the intervention is classified as “Very Cost-Effective”; (b) if DAH/DALYs averted = 1×–3× GDP per capita, then the intervention is classified as “Cost-Effective”; (c) if DAH/DALYs averted > 3× GDP per capita, then the intervention is classified as “Non-Cost-Effective”. Finally, we adopted a dominance framework for evaluation: countries with improved outcomes are classified as Dominant, while those with worse outcomes despite increased funding are marked as Dominated.

## 3. Results

This section presents the findings of our analysis on the cost-effectiveness of DAH in Sub-Saharan Africa, focusing on HIV/AIDS, malaria, and tuberculosis between 1995 and 2018. The results are structured in two main parts: (a) the effectiveness of DAH by disease and (b) the efficiency of DAH at the country level. Through regression analysis, Z-score normalization, and quadrant matrix visualizations, we highlight variations in how resources are absorbed and converted into health outcomes, as measured by averted DALYs. The GCEA framework is used to classify interventions as very cost-effective, cost-effective, or non-cost-effective. Table 1 presents the regression results of DAH on averted DALYs for the three diseases, disaggregated by HDI classification.

The regression analysis indicates that Development Assistance for Health (DAH) impacts DALYs differently across countries with varying HDI levels. In HIV/AIDS, DAH has a positive but not significant effect in low-HDI countries, while it is slightly negative and marginally substantial in middle/high-HDI countries. The number of physicians significantly reduces HIV/AIDS DALYs in middle/high-HDI nations. For malaria, DAH significantly benefits low-HDI countries but not middle/high-HDI ones. In tuberculosis, DAH only shows a significant positive effect in low-HDI countries, with physicians and immunizations negatively associated in respective contexts. Model fit is moderate to strong, particularly in low-HDI countries. Overall, DAH and health system factors have a greater impact in less developed areas. All models use robust OLS with heteroskedasticity-consistent errors, ensuring reliable estimates.

To assess the robustness of our findings, we conducted sensitivity analyses by varying the threshold values defining HDI groups. To ensure that our results are not sensitive to this categorization, we conducted sensitivity analyses by varying the cutoff between low- and middle/high-HDI groups (e.g., 0.50 and 0.60 thresholds). The results were broadly consistent with our main findings: the positive association between total DAH and averted DALYs remained robust in the low-HDI group across all cutoffs, while the effect in the middle/high-HDI group remained weak or non-significant. These sensitivity checks confirm that our conclusions are not sensitive to the specific HDI cutoff chosen, supporting the validity of the threshold used in the main analysis (Appendix A).

In terms of aggregate DALYs averted and funding levels (Table 2), HIV/AIDS received the highest total DAH, but its cost-effectiveness ratio ($7.50 per DALY averted) was the least favorable. Tuberculosis, while underfunded, showed a highly efficient return at just $1.02 per DALY averted. Malaria had a balanced profile, with a moderate funding level and strong outcomes ($1.80 per DALY averted).

**Figure 1:**
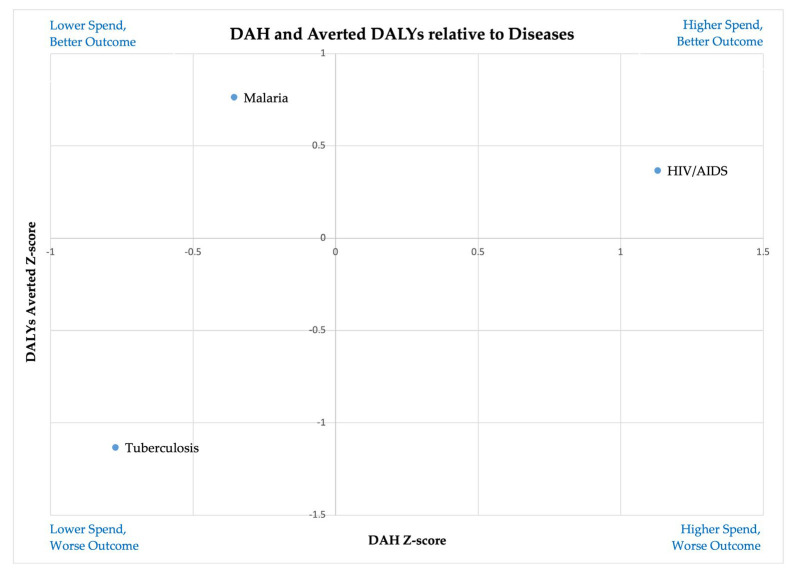
Mapping of diseases according to DAH and DALYs averted.

Τhe Z-Score index was used to describe and graphically depict, through quadrant matrix charts, the position of values within the overall dataset of DAH and DALYs averted in relation to their mean and standard deviation. The results are presented in Figure 1.

The quadrant matrix chart reveals that HIV/AIDS is positioned in the top-right quadrant, signifying above-average funding and strong health outcomes, suggesting an effective allocation of resources. Malaria appears in the top-left quadrant, demonstrating that despite receiving below-average funding, it achieves relatively high DALYs averted, indicating high cost-effectiveness. In contrast, tuberculosis falls in the bottom-left quadrant, reflecting both low DAH spending and poor health outcomes, which suggests underfunding or inefficiencies in intervention strategies. Notably, no disease appears in the bottom-right quadrant, which would indicate high spending with poor outcomes.

**Figure 2:**
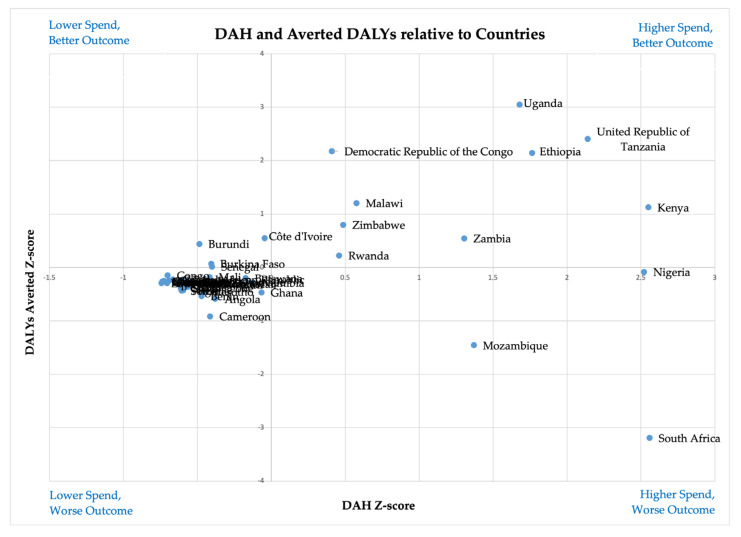
Mapping of countries by DAH and DALYs averted.

To assess country-level cost-effectiveness, we calculated DAH-to-DALYs averted ratios per country (see Table S1 in the Supplementary File) and visualized these using Z-score-based quadrant matrices (Figure 2).

Several countries, with the most prominent being Uganda, Ethiopia, and the United Republic of Tanzania, are positioned in the top-right quadrant, indicating high DAH spending with strong health outcomes, suggesting effective resource utilization. Conversely, Mozambique and South Africa are in the bottom-right quadrant, signifying high DAH spending but poor health outcomes, indicating potential inefficiencies in resource allocation. The majority of the countries cluster at the bottom-left quadrant but near the center, which suggests relatively balanced DAH spending and health outcomes.

To refine our understanding, we expanded the analysis to examine DAH and averted DALYs per disease and country, with the corresponding Z-scores (Tables S2–S4 in the Supplementary File). This multidimensional mapping enables us to identify countries that are particularly effective (or ineffective) in using DAH for specific diseases. For HIV/AIDS, countries such as Uganda, Tanzania, Kenya, Ethiopia, and Zimbabwe stand out for achieving strong health outcomes relative to their funding levels. These countries combine substantial DAH with above-average performance in terms of DALYs averted, indicating an efficient and impactful use of resources. Conversely, South Africa, Mozambique, and Nigeria received high HIV-related DAH but achieved comparatively poor results in DALYs averted. These cases suggest inefficiencies in converting investment into health outcomes, potentially due to systemic health infrastructure challenges or program delivery issues. Some countries, including Burundi and Rwanda, achieved positive results despite more modest DAH levels, signaling high efficiency and cost-effectiveness in their HIV/AIDS programs.

Regarding malaria, high-performing countries in terms of both funding and outcomes include Uganda, Tanzania, Mozambique, Malawi, and Nigeria, as they demonstrate strong absorption and deployment of DAH with tangible health impacts. Kenya, Rwanda, and Burundi also show strong results, despite receiving relatively moderate levels of malaria-related DAH. On the other end of the spectrum, countries such as Cameroon, Benin, and Niger show poor health outcomes alongside limited funding, suggesting underinvestment and potential barriers to effective malaria control.

For tuberculosis, Ethiopia, the Democratic Republic of the Congo, Zambia, and Uganda emerge as leading examples of efficient DAH use. These countries achieved high levels of DALYs averted and received substantial but not excessive funding, making them exemplary cases of cost-effectiveness. In contrast, South Africa, despite receiving one of the highest allocations of tuberculosis-related DAH, did not perform proportionately well in terms of health outcomes. This pattern mirrors its HIV/AIDS profile and may indicate broader systemic issues affecting health intervention efficiency. Other countries such as Burkina Faso, Guinea, and Cameroon reported low performance both in funding and outcomes, highlighting the need for increased support or programmatic reevaluation.

**Figure 3:**
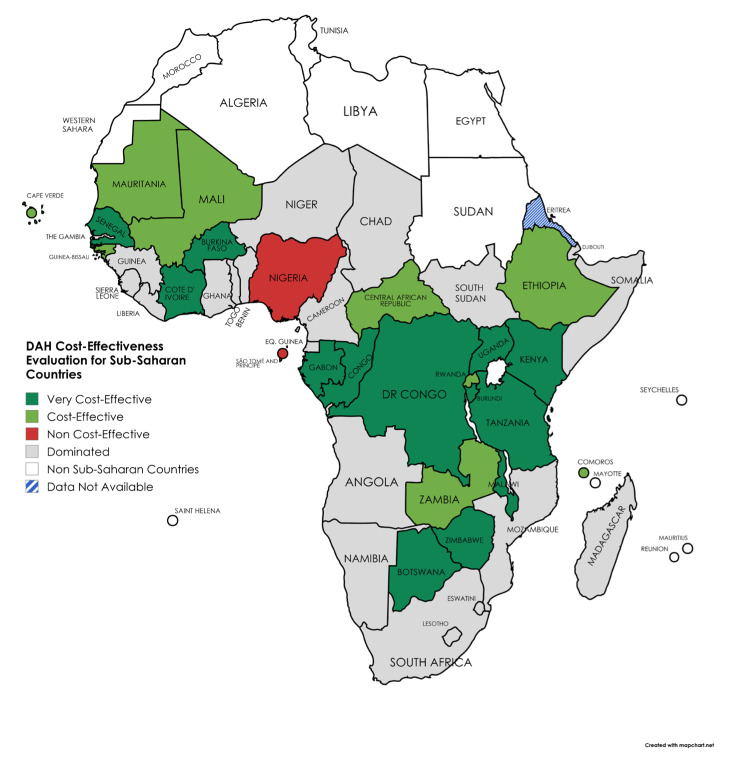
Cost-effectiveness ratio for all the reference countries.

Finally, the cost-effectiveness ratio for each country is calculated and the results are presented in Figure 3 (for the detailed results see Table S5 in Supplementary File).

According to our analysis, 13 countries were classified as very cost-effective recipients of DAH, 9 countries were considered cost-effective, and 2 countries were identified as non-cost-effective recipients of DAH. Among the low-HDI countries, 8 were found to be very cost-effective and 6 cost-effective, while 1 was non–cost-effective. In the medium-HDI group, 3 countries were very cost-effective and 3 were cost-effective, with 1 identified as non–cost-effective. For countries in the high HDI group, 2 were classified as very cost-effective and 1 as dominated. Both low- and medium-HDI groups included a substantial proportion of countries (approximately 50%, 14 and 6, respectively) where the cost-effectiveness result was dominated. In total, 21 countries demonstrated no reduction in DALYs for the diseases examined, thus identifying as dominated in the framework of the analysis. These results indicate considerable variation in performance within each HDI category, suggesting that DAH effectiveness is significantly context-dependent, potentially influenced by health system capacity and absorption efficiency. Future aid targeting health system strengthening in those countries should increase, as this approach would also benefit the cost-effectiveness of other targeted interventions, those against communicable diseases included.

The results presented above highlight the complex and diverse landscape of DAH effectiveness across Sub-Saharan Africa. While certain countries and diseases show a clear alignment between investment and impact, identifying them as strong candidates for continued or increased support, others expose inefficiencies that deserve closer examination.

## 4. Discussion

The cost-effectiveness of DAH has been extensively studied since the early 2000s. According to Neumann et al. [18], 479 studies estimated cost per DALY averted between 2000 and 2015, with a peak of nearly 90 in a single year. In Sub-Saharan Africa, Cost-Effectiveness Analysis has become the dominant framework for evaluating health interventions, particularly those targeting communicable diseases [19].

As early as 1999, Goodman et al. [20] emphasized that the cost of essential malaria interventions exceeded the financial capacity of low-income countries, making external assistance indispensable. This observation has been reinforced by subsequent studies [21,22], which demonstrate that development aid contributes to improved health outcomes by increasing available resources and strengthening healthcare delivery systems. Our findings support this perspective: Since the mid-2000s, DAH has risen sharply, while DALYs attributable to malaria have steadily declined—indicating the effective use of aid in combating the disease.

A similar pattern is observed for tuberculosis. Baltussen et al. [23] also stressed that critical tuberculosis interventions are financially out of reach for many low-income nations. Our results align with Ralaidovy et al. [24], who concluded that DAH-funded interventions for all three diseases—HIV, malaria, and tuberculosis—are broadly cost-effective. However, both their study and ours highlight the importance of complementing financial aid with technical support to maximize impact.

On the other hand, our analysis shows that DAH allocations for HIV/AIDS, while substantial, yield lower cost-effectiveness compared to malaria and tuberculosis. This finding is consistent with Yogo and Mallaye [25], who recognized DAH’s positive role in HIV management but argued it remains insufficient. Crucially, our study does not suggest that funding for HIV should be reduced; on the contrary, we emphasize that any reduction could lead to severe setbacks. Recognizing disease-specific constraints is vital for maximizing the long-term effectiveness of global health aid. HIV/AIDS, which in the Sub-Saharan context is predominantly sexually transmitted, poses unique challenges in terms of prevention: Behavioral change interventions often encounter barriers rooted in illiteracy, cultural norms, and stigma [26], which complicate the effectiveness of aid-financed programs. These factors may partly explain the relatively lower cost-effectiveness observed for HIV/AIDS interventions compared to malaria and tuberculosis. DAH interventions should be tailor-made to the diseases’ context, and in the case of HIV/AIDS, these should include investing in community-based education, behavioral interventions, and stigma reduction. At the same time, adherence, as well as effective adherence monitoring, are vital for optimizing interventions’ effectiveness [27].

Nonetheless, our findings also raise the possibility of ceiling effects in some countries. In regions where the level of communicable diseases—such as HIV/AIDS, malaria, or tuberculosis—is already relatively low, further Development Assistance for Health (DAH) may produce limited additional benefits. This is not necessarily due to ineffective aid use, but rather the natural decline in health improvements as disease control approaches its maximum. Therefore, cost-effectiveness ratios might seem unfavorable, even if aid is effectively targeted and properly implemented [28,29]

A clear example is South Africa, which received some of the highest DAH levels for HIV/AIDS during the study period. Despite this, its DALYs averted per dollar was relatively weak, especially compared to lower-HDI countries like Uganda or Ethiopia, which saw better health outcomes with less funding. This seeming inefficiency may partly result from a ceiling effect: South Africa’s advanced health infrastructure and lower initial disease burden make large DALY reductions more challenging, even with significant financial investment.

This observation aligns with the broader literature warning that uniform cost-effectiveness thresholds might overlook baseline burden and contextual factors [30,31]. In these situations, ongoing investment remains beneficial, not necessarily for reducing DALYs, but also for maintaining control, preventing resurgence, and tackling structural health disparities. Future DAH evaluations may benefit from adjusting benchmarks to reflect context-sensitive ceilings in achievable impact.

Furthermore, the growing burden of NCDs in low-income countries must be acknowledged. Ruby et al. [7] and the Global Burden of Disease Collaborative Network [12] projected that NCDs will become the leading cause of mortality in Sub-Saharan Africa by 2030. Addressing this shift will require substantial institutional responses at both national and international levels, including not only financial aid but also the development of appropriate infrastructure and policy support [19,32].

Finally, regarding the methodological approach of the study, we employed GDP per capita as a threshold for evaluating cost-effectiveness—still the most widely used benchmark [33,34]. Nevertheless, this approach is not without limitations. Revill et al. [35] argue that GDP-based thresholds may overlook country-specific constraints and context, potentially undervaluing necessary interventions in low-resource settings. Furthermore, the upper threshold of 3× GDP per capita may classify interventions as cost-effective even when their real-world utility is questionable. While some propose that each country should develop its own customized thresholds, such efforts are complex and rarely implemented [33].

Microinsurance has emerged as a critical tool for enhancing financial inclusion and reducing vulnerability among low-income populations. The fundamental concept of microinsurance, alongside other microfinance services, is providing households with the necessary funding to acquire essential assets and manage exposure to risks. According to Platteau et al. [36], these insurance programs offer protection against the financial consequences of life-cycle risks, helping households escape poverty traps. Also, microinsurance offers affordable, tailored risk management solutions, enabling individuals and families to safeguard their livelihoods against unforeseen events such as crop failures, illness, and natural disasters [37]. Microinsurance is a vital financial tool by offering risk coverage that exceeds the protection of microloans and precautionary savings, especially during extreme shocks. However, its adoption faces significant barriers. A key challenge lies in communicating its value effectively, as many struggle to perceive the long-term benefits of paying premiums compared to immediate financial needs. Addressing this reluctance requires innovative strategies to enhance understanding and trust, ensuring that microinsurance is seen as an accessible and essential safety net rather than an unnecessary expense.

The low-income countries offer a fertile ground for microinsurance where risk mechanisms and insurance markets are imperfect. While individuals are characterized by higher risk aversion, as the disposal income is lower, we would expect the demand for these products to be significantly higher—nonetheless, the empirical evidence casts doubt upon this hypothesis. McCord et al. [38], studying insurance coverage in Africa, mentioned the low rates in health and agriculture products. Among various factors affecting microinsurance adoption, institutional structure and policy levels affect the demand side. For example, in Bangladesh, the demand for health microinsurance is boosted by the absence of a governmental plan to offer health services to the poor [39]. In the same line, Churchill & Matul [40] argue that regulatory frameworks play a critical role.

Many low-income countries face obstacles in achieving universal health coverage due to particularly limited public resources. As a result, access to healthcare facilities and the burden of cost are unequally distributed, especially among people experiencing poverty. Despite its potential, the adoption of microinsurance remains constrained by factors such as limited awareness, affordability issues, and trust in insurance providers [41]. As Insurance and Risk Finance Facility [42] mentioned, globally, 15% of that market is currently reached, and just 11.5% of the target population is covered (330 million people) by a microinsurance product. In Africa, 9.4% of the population is covered, while the estimated value of the microinsurance market is 6.9 billion. The low coverage rates highlight the significant protection gap and then emerge to take action to close it. The literature suggests that community-based health insurance or “mutual health organizations” have become increasingly popular models delivering microinsurance. These organizations have great coverage in the health insurance market due to the low interest of commercial insurers in entering the market. Several studies claim that community-based insurance is a prominent model where members pool resources to cover healthcare costs. For example, Rwanda’s Mutuelles de Santé scheme has dramatically increased healthcare coverage. The government’s target policy for premiums resulted in over 90% enrollment, premium subsidization for the poorest citizens by the government and donors, and higher contributions for wealthier participants. Lu et al. [43] concluded that the Mutuelles de Santé scheme has significantly improved healthcare utilization and financial protection despite the challenges. Additionally, provider-driven microinsurance schemes, often led by healthcare providers or facilities, aim to ensure affordable access to their services, such as the Kilifi Health Insurance Scheme in Kenya, which subsidizes care at local clinics. Finally, private microinsurance initiatives, often supported by partnerships between insurers and mobile technology companies, are gaining traction, offering innovative products such as mobile-based health insurance plans. Each model addresses specific barriers to healthcare, but their success often depends on factors such as affordability, trust, and the availability of healthcare infrastructure.

Microinsurance is indispensable in advancing healthcare access and financial security in Sub-Saharan Africa, particularly for low-income populations. Programs like Rwanda’s Mutuelles de Santé demonstrate how well-designed policies can extend coverage to vulnerable groups and foster long-term socio-economic development. However, the sustainability of these schemes requires addressing operational challenges, improving infrastructure, and encouraging greater collaboration among governments, private insurers, and NGOs. By building resilient microinsurance systems, Sub-Saharan Africa can make significant strides toward achieving universal health coverage and improving overall quality of life.

## 5. Conclusions

Using tools from health economics and statistical analysis, this study assessed the cost-effectiveness of DAH in Sub-Saharan Africa between 1995 and 2018, focusing on HIV/AIDS, malaria, and tuberculosis. We examined the efficiency of DAH allocation across diseases, individual countries, and groups categorized by HDI, offering a multidimensional view of health aid absorption and impact.

Our findings indicate that DAH directed toward malaria and tuberculosis was significantly associated with reductions in DALYs, particularly in low-HDI countries. For HIV/AIDS, a significant effect emerged primarily in middle/high-HDI countries, while overall efficiency was lower compared to the other two diseases. When evaluating interventions using GCEA, 13 countries were classified as very cost-effective recipients of DAH, 9 as cost-effective, and 2 as non-cost-effective. However, 21 countries showed no DALYs reduction in the diseases in question, highlighting inefficiencies in either fund utilization or health system capacity.

This three-pronged approach—analyzing outcomes by country, disease, and HDI level—provides a practical foundation for policymakers and donor organizations to make more targeted, evidence-based funding decisions. While DAH has played a vital role in advancing communicable disease outcomes across the region, its current form, primarily focused on disease-specific interventions, may not be sufficient to drive lasting improvements. Therefore, a shift towards system-level investments is essential. Strengthening health systems holistically—through better infrastructure, human resources, information systems, and accountability mechanisms—can increase the long-term effectiveness of targeted aid. This includes improving data systems for more accurate epidemiological monitoring and outcome tracking, a concern echoed widely in the international literature.

In this context, microinsurance has become vital for promoting financial inclusion and protecting low-income populations in Sub-Saharan Africa from life-cycle risks such as illness and natural disasters. Although it offers more comprehensive protection than microloans or savings, its adoption is hindered by low awareness, affordability issues, and mistrust. While demand might be expected to be high due to greater risk aversion among poorer households, empirical evidence shows low uptake, particularly in health and agriculture insurance. Community-based health insurance models, such as Rwanda’s successful Mutuelles de Santé and provider-driven or mobile-based schemes, have effectively addressed these gaps. Despite challenges, microinsurance remains essential for improving healthcare access, financial resilience, and long-term development in the region. Sustainable success depends on supportive policies, infrastructure improvements, and collaboration between the public and private sectors.

Although countries in Sub-Saharan Africa differ in how efficiently they utilize health aid, they share a common challenge: a continued reliance on external support without achieving relative self-sufficiency. Going forward, DAH must not only address current health burdens but also lay the groundwork for resilient, self-sustaining systems. Only then can aid truly empower countries to lead their own health development agendas.

## Figures and Tables

**Table 1:** Regression analysis of DAH on averted DALYs per HDI group.

Variable	Low HDI	Middle/High HDI
	**HIV/AIDS**	
Total DAH	0.23 (0.25)	−0.03 (0.21) *
Physicians	−1,593,008 (2,417,011)	−7,440,497 (3,654,346) *
DPT Immunization	14,991(14,483.1)	31,602.02 (23,008)
Constant	−457,977 (666,196.9)	−682,966 (1,398,320)
R-squared	0.65	0.71
	**Malaria**	
Total DAH	0.98 (0.30) ***	0.32 (0.22)
Physicians	962,196.5 (728914) *	331,209.6 (326,599)
DPT Immunization	4379.7 (4553)	1110.6 (4082.95)
Constant	−439,582.6 (369671)	−268,411 (275,339)
R-squared	0.83	0.58
	**Tuberculosis**	
Total DAH	2.82 (1.11) **	0.02 (0.28)
Physicians	−224,393 (671,796) *	40,710.5 (253,495)
DPT Immunization	−1004.9 (3328)	−6066.35 (3338) *
Constant	−42,930.06 (299,462)	456,983.8 (271,707)
R-squared	0.74	0.54

Note: Standard errors are robust and shown in parentheses; significance: *** *p* < 0.01, ** *p* < 0.05, * *p* < 0.1; coefficients and SEs rounded for readability.

**Table 2:** DAH and averted DALYs per disease.

Diseases	Total DAH (in thousand $)	Aggregate Averted DALYs	DAH Z-Score	DALYs Averted Z-Score	DAH to Averted DALYs (in $)
Tuberculosis	$4,688,983	4,616,429	−0.773	−1.132	$1.02
HIV/AIDS	$68,552,349	9,139,928	1.129	0.368	$7.50
Malaria	$18,650,005	10,334,075	−0.357	0.764	$1.80
Total	$91,891,337	24,090,433	-	-	$3.81

**Figure A1:**
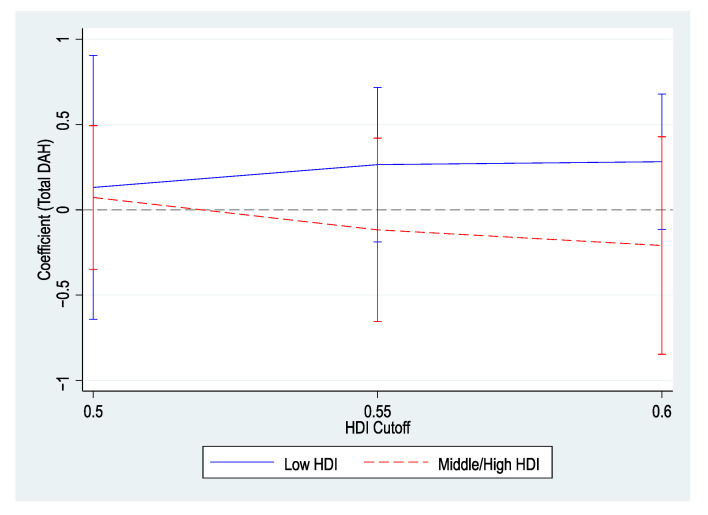
Sensitivity Analysis: Coefficient of Total DAH by HDI Cutoff.

## Data Availability

The original contributions presented in this study are included in the article/Supplementary Materials. Further inquiries can be directed to the corresponding author.

## Supplementary Materials

The following supporting information can be downloaded at: https://www.mdpi.com/article/10.3390/healthcare13141716/s1, Table S1: DAH to Averted DALYs (per country); Table S2: Z-scores for DAH and averted DALYs for HIV/AIDS; Table S3: Z-scores for DAH and averted DALYs for Malaria; Table S4: Z-scores for DAH and averted DALYs for Tuberculosis; Table S5: Cost-Effectiveness ratio for all Sub-Saharan countries.

## Abbreviations

The following abbreviations are used in this manuscript:

DAH	Development Assistance for Health
IHME	Institute for Health Metrics and Evaluation
GCEA	Generalized Cost-Effectiveness Analysis
DALYs	Disability-Adjusted Life Years
HDI	Human development index
NCDs	Non-Communicable Diseases
GDP	Gross Domestic Product

## References

[1] Gorodensky A., Bowra A., Saeed G., Kohler J.C. (2022). Anti-corruption in global health systems: Using key informant interviews to explore anti-corruption, accountability and transparency in international health organisations. BMJ Open.

[2] Moitra M., Cogswell I.E., Maddison E.R., Simpson K., Stutzman H.N., Tsakalos G., Dieleman J., Micah A.E. (2021). Factors associated with the disbursements of development assistance for health in low-income and middle-income countries, 2002–2017. BMJ Glob. Health.

[3] Xie S., Du S., Huang Y., Luo Y., Chen Y., Zheng Z., Yuan B., Xu M., Zhou S. (2025). Evolution and effectiveness of bilateral and multilateral development assistance for health: A mixed-methods review of trends and strategic shifts (1990–2022). BMJ Glob. Health.

[4] Institute for Health Metrics and Evaluation (2020). Financing Global Health 2020: The Impact of COVID-19.

[5] Shi J., Jin Y., Zheng Z. (2023). Addressing global health challenges requires harmonised and innovative approaches to the development assistance for health. BMJ Glob. Health.

[6] Radelet S. (2006). A Primer on Foreign Aid. Center for Global Development. Working Paper No. 92. https://www.cgdev.org/publication/primer-foreign-aid-working-paper-92.

[7] Ruby A., Knight A., Perel P., Blanchet K., Roberts B. (2015). The effectiveness of interventions for non-communicable diseases in humanitarian crises: A systematic review. PLoS ONE.

[8] Holmes M.D., Dalal S., Volmink J., Adebamowo C.A., Njelekela M., Fawzi W.W., Willett W.C., Adami H.-O. (2010). Non-communicable diseases in sub-Saharan Africa: The case for cohort studies. PLoS Med..

[9] Heestermans T., Browne J.L., Aitken S.C., Vervoort S.C., Klipstein-Grobusch K. (2016). Determinants of adherence to antiretroviral therapy among HIV-positive adults in sub-Saharan Africa: A systematic review. BMJ Glob. Health.

[10] Bijker R., Jiamsakul A., Kityo C., Kiertiburanakul S., Siwale M., Phanuphak P., Akanmu S., Chaiwarith R., Wit F.W., Sim B.L. (2017). Adherence to antiretroviral therapy for HIV in sub-Saharan Africa and Asia: A comparative analysis of two regional cohorts. J. Int. AIDS Soc..

[11] World Health Organization (2021). World Health Statistics 2021: Monitoring Health for the SDGs, Sustainable Development Goals.

[12] Global Burden of Disease Collaborative Network (2021). Global Health Spending 1995–2018.

[13] Institute for Health Metrics and Evaluation (2021). Financing Global Health Visualization.

[14] United Nations Development Programme (2021). Human Development Indicators. http://hdr.undp.org.

[15] Rubinstein A., García Martí S., Souto A., Ferrante D., Augustovski F. (2009). Generalized cost-effectiveness analysis of a package of interventions to reduce cardiovascular disease in Buenos Aires, Argentina. Cost Eff. Resour. Alloc..

[16] Ochalek J., Revill P., Drummond M., Revill P., Suhrcke M., Moreno-Sera R., Sculpher M. (2020). Allocating scarce resources—Tools for priority setting. Global Health Economics: Shaping Health Policy in Low- and Middle-Income Countries.

[17] Edejer T.T.T., Baltussen R., Adam T., Hutubessy R., Acharya A., Evans D.B., Murray C.J.L. (2017). Making Choices in Health: WHO Guide to cost-Effectiveness Analysis.

[18] Neumann P.J., Thorat T., Zhong Y., Anderson J., Farquhar M., Salem M., Sandberg E., Saret C.J., Wilkinson C., Cohen J.T. (2016). A systematic review of cost-effectiveness studies reporting cost-per-DALY averted. PLoS ONE.

[19] Panzer A.D., Emerson J.G., D’CRuz B., Patel A., Dabak S., Isaranuwatchai W., Teerawattananon Y., Ollendorf D.A., Neumann P.J., Kim D.D. (2020). Growth and capacity for cost-effectiveness analysis in Africa. Health Econ..

[20] Goodman C.A., Coleman P.G., Mills A.J. (1999). Cost-effectiveness of malaria control in sub-Saharan Africa. Lancet.

[21] Schmidt A. (2009). Health aid Effectiveness in Nepal: Paris, Accra, Civil Society and the Poor.

[22] Mishra P., Newhouse D. (2009). Does health aid matter?. J. Health Econ..

[23] Baltussen R., Floyd K., Dye C. (2005). Cost effectiveness analysis of strategies for tuberculosis control in developing countries. BMJ.

[24] Ralaidovy A.H., Lauer J.A., Pretorius C., Briët O.J., Patouillard E. (2021). Priority setting in HIV, tuberculosis, and malaria—New cost-effectiveness results from WHO-CHOICE. Int. J. Health Policy Manag..

[25] Yogo U.T., Mallaye D. (2015). Health aid and health improvement in Sub-Saharan Africa: Accounting for the heterogeneity between stable states and post-conflict states. J. Int. Dev..

[26] UNAIDS (2024). The Urgency of Now: AIDS at a Crossroads.

[27] Haberer J.E., Sabin L., Amico K.R., Orrell C., Galárraga O., Tsai A.C., Vreeman R.C., Wilson I., Sam-Agudu N.A., Blaschke T.F. (2017). Improving antiretroviral therapy adherence in resource-limited settings at scale: A discussion of interventions and recommendations. J. Int. AIDS Soc..

[28] Nugent R., Bertram M.Y., Jan S., Niessen L.W., Sassi F., Jamison D.T., Pier E.G., Beaglehole R. (2018). Investing in non-communicable disease prevention and management to advance the Sustainable Development Goals. Lancet.

[29] Ravallion M. (2020). Should the Randomistas (Continue to) Rule?.

[30] Neumann P.J., Sanders G.D., Russell L.B., Siegel J.E., Ganiats T.G. (2016). Cost-Effectiveness in Health and Medicine.

[31] Revill P., Ochalek J., Lomas J., Nakamura R., Woods B., Rollinger A., Suhrcke M., Sculpher M., Claxton K. (2017). Cost-effectiveness thresholds: Guiding health care spending for population health improvement. F1000Research.

[32] Owolade A., Mashavakure H., Babatunde A.O., Aborode A.T. (2022). Time to relook into non-communicable diseases (NCDs) in Africa: A silent threat overwhelming global health in Africa. Ann. Med. Surg..

[33] Leech A.A., Kim D.D., Cohen J.T., Neumann P.J. (2018). Use and misuse of cost-effectiveness analysis thresholds in low- and middle-income countries: Trends in cost-per-DALY studies. Value Health.

[34] Kazibwe J., Gheorghe A., Wilson D., Ruiz F., Chalkidou K., Chi Y.L. (2022). The use of cost-effectiveness thresholds for evaluating health interventions in low- and middle-income countries from 2015 to 2020: A review. Value Health.

[35] Revill P., Walker S.M., Madan J., Ciaranello A., Mwase T., Gibb D.M., Claxton K., Sculpher M.J. (2014). Using Cost-Effectiveness Thresholds to Determine Value for Money in Low- and Middle-Income Country Healthcare Systems: Are Current International Norms Fit for Purpose?.

[36] Platteau J.P., De Bock O., Gelade W. (2017). The demand for microinsurance: A literature review. World Dev..

[37] Oppong E.O., Yu B., Mazonga Mfoutou B.O. (2024). The effect of microinsurance on the financial resilience of low-income households in Ghana: Evidence from a propensity score matching analysis. Geneva Pap. Risk Insur. Issues Pract..

[38] McCord M.J., Opdebeeck B., Biese K., Pandey M. (2014). ‘If you can’t measure it, you can’t manage it’: Microinsurance by the numbers. Enterp. Dev. Microfinance.

[39] Hamid S.A., Roberts J., Mosley P. (2011). Can micro health insurance reduce poverty? Evidence from Bangladesh. J. Risk Insur..

[40] Churchill C., Matul M. (2012). Protecting the Poor—A Microinsurance Compendium.

[41] Banerjee A., Breza E., Duflo E., Kinnan C. (2019). Can Microfinance Unlock a Poverty Trap for Some Entrepreneurs?.

[42] Insurance and Risk Finance Facility (2024). Almost 90% of People in Low-Income Countries Have no Access to Insurance, Reveals New Study. United Nations Development Programme [Internet]..

[43] Lu C., Chin B., Lewandowski J.L., Basinga P., Hirschhorn L.R., Hill K., Murray M., Binagwaho A., Postma M. (2012). Towards universal health coverage: An evaluation of Rwanda Mutuelles in its first eight years. PLoS ONE.

