# Supplementary material for: From Aid to Impact: The Cost-Effectiveness of Global Health Aid in Sub-Saharan Africa and the Evolving Role of Microinsurance
Source: Healthcare (Basel). 2025 Jul 16;13(14):1716. doi: 10.3390/healthcare13141716 (PMC12294523; doi:10.3390/healthcare13141716)
Supplement: Supplementary file 1 [file healthcare-13-01716-s001.zip › healthcare-3728770-supplementary.pdf]

Supplementary file: Annex

Table S1. DAH to Averted DALYs (per country)

Country	Total DAH (in thousand $)	Aggregate Averted DALYs	DAH to averted DALYs (in $)
Namibia	1657936	-33,959	-48.82
Liberia	499459	-15,919	-31.38
Gambia	260040	-13,101	-19.85
Togo	361871	-19,191	-18.86
South Sudan	570904	-34,486	-16.55
Madagascar	821932	-72,102	-11.40
Eswatini	908897	-102,516	-8.87
Ghana	1817737	-393,681	-4.62
Guinea	488763	-194,032	-2.52
Djibouti	115573	-46,270	-2.50
Mozambique	5645772	-2,340,271	-2.41
Lesotho	805895	-364,672	-2.21
Sierra Leone	402250	-235,558	-1.71
Angola	985221	-629,842	-1.56
South Africa	8813330	-5,758,885	-1.53
Chad	366885	-241,942	-1.52
Benin	737578	-524,958	-1.41
Niger	408913	-314,946	-1.30
Somalia	378695	-326,432	-1.16
Cameroon	894105	-1,277,749	-0.70
Equatorial Guinea	17595	-50,914	-0.35
Nigeria	8714566	360,953	24.14
Botswana	1534050	136,222	11.26
Sao Tome and Principe	39145	3,935	9.95
Cabo Verde	31182	4,225	7.38
Mauritania	49602	8,728	5.68
Eritrea	410244	73,592	5.57
Comoros	39991	7,722	5.18
Mali	882480	177,264	4.98
Zambia	5471778	1,597,602	3.42
Rwanda	3217631	965,708	3.33
Guinea-Bissau	149815	45,525	3.29
Kenya	8791218	2,744,596	3.20
Central African Republic	223433	84,798	2.63
Gabon	59065	28,567	2.07
Senegal	928677	560,024	1.66
Zimbabwe	3291425	2,100,432	1.57
United Republic of Tanzania	7697182	5,271,191	1.46
Ethiopia	6695879	4,749,801	1.41
Burkina Faso	913560	669,156	1.37
Malawi	3532978	2,900,079	1.22
Côte d'Ivoire	1875843	1,612,496	1.16
Uganda	6468860	6,532,210	0.99
Democratic Republic of the Congo	3087237	4,811,759	0.64

Congo	127686	239,503	0.53
Burundi	698459	1,395,772	0.50

Table S2. Z-scores for DAH and averted DALYs for HIV/AIDS

Diseases	Country	Total DAH z-score	Aggregate Averted DALYs z-score
hiv/aids	Angola	-0,4798944	-0,663259
hiv/aids	Benin	-0,5317369	-0,1463241
hiv/aids	Botswana	0,00103654	-0,0433444
hiv/aids	Burkina Faso	-0,4655617	0,43156358
hiv/aids	Burundi	-0,5005576	0,3735247
hiv/aids	Cabo Verde	-0,6651438	-0,1262648
hiv/aids	Cameroon	-0,4114799	-0,6224466
hiv/aids	Central African Republic	-0,6208682	0,04353012
hiv/aids	Chad	-0,5935836	-0,1918952
hiv/aids	Comoros	-0,6718561	-0,1277525
hiv/aids	Congo	-0,6363198	-0,0286756
hiv/aids	Côte d'Ivoire	-0,0170627	0,8325785
hiv/aids	Democratic Republic of the Congo	-0,0912764	0,83381521
hiv/aids	Djibouti	-0,6398623	-0,1544156
hiv/aids	Equatorial Guinea	-0,674215	-0,196687
hiv/aids	Eritrea	-0,5632906	-0,0928458
hiv/aids	Eswatini	-0,3041171	-0,192826
hiv/aids	Ethiopia	1,54568122	1,52707226
hiv/aids	Gabon	-0,663104	-0,1352531
hiv/aids	Gambia	-0,6289431	-0,1573076
hiv/aids	Ghana	-0,2879455	-0,2531053
hiv/aids	Guinea	-0,571511	-0,2264111
hiv/aids	Guinea-Bissau	-0,6435321	-0,1507409
hiv/aids	Kenya	2,64610839	1,43072815
hiv/aids	Lesotho	-0,3339987	-0,3305404
hiv/aids	Liberia	-0,5903211	-0,1620755
hiv/aids	Madagascar	-0,6123151	-0,2138365
hiv/aids	Malawi	0,580345	0,98499374
hiv/aids	Mali	-0,4831846	-0,1992222
hiv/aids	Mauritania	-0,6680014	-0,1265541
hiv/aids	Mozambique	1,4155104	-2,1994699
hiv/aids	Namibia	0,02361503	-0,1663988
hiv/aids	Niger	-0,603557	-0,1194584
hiv/aids	Nigeria	2,11742064	-1,4131657
hiv/aids	Rwanda	0,44585401	0,05093412
hiv/aids	Sao Tome and Principe	-0,6738591	-0,1276705
hiv/aids	Senegal	-0,5001999	-0,1368563
hiv/aids	Sierra Leone	-0,5753257	-0,2033954
hiv/aids	Somalia	-0,6209368	-0,2181909
hiv/aids	South Africa	3,06192599	-3,8716833
hiv/aids	South Sudan	-0,5786964	-0,2307767
hiv/aids	Togo	-0,5829571	-0,1391209
hiv/aids	Uganda	1,64066031	2,66237114
hiv/aids	United Republic of Tanzania	2,04202569	2,28290026
hiv/aids	Zambia	1,37997278	0,57582929

hiv/aids	Zimbabwe	0,58505877	1,33812889

Table S3. Z-scores for DAH and averted DALYs for Malaria

Diseases	Country	Total DAH z-score	Aggregate Averted DALYs z-score
malaria	Angola	0,21542108	-0,693511
malaria	Benin	-0,0527574	-1,1689648
malaria	Botswana	-0,826528	-0,3602675
malaria	Burkina Faso	-0,0423261	-0,4971019
malaria	Burundi	-0,287857	0,52653489
malaria	Cabo Verde	-0,8314554	-0,3611005
malaria	Cameroon	-0,2825102	-1,1114259
malaria	Central African Republic	-0,7002658	-0,4841485
malaria	Chad	-0,4886278	-0,4335773
malaria	Comoros	-0,7836851	-0,3575809
malaria	Congo	-0,7802101	-0,2955089
malaria	Côte d'Ivoire	-0,0723783	-0,2311633
malaria	Democratic Republic of the Congo	2,24590454	4,14598151
malaria	Djibouti	-0,7982629	-0,3670348
malaria	Equatorial Guinea	-0,8171028	-0,3183192
malaria	Eritrea	-0,5775406	-0,3572554
malaria	Eswatini	-0,7654071	-0,3565875
malaria	Ethiopia	2,07032256	-0,8857063
malaria	Gabon	-0,7847326	-0,3137392
malaria	Gambia	-0,5961327	-0,2976131
malaria	Ghana	0,89738018	-0,6466187
malaria	Guinea	-0,3609649	-0,4647232
malaria	Guinea-Bissau	-0,7234816	-0,248214
malaria	Kenya	1,73861837	0,50666531
malaria	Lesotho	-0,8369778	-0,3608238
malaria	Liberia	-0,2687898	-0,3435171
malaria	Madagascar	0,48807043	-0,2505699
malaria	Malawi	0,32983881	1,24669698
malaria	Mali	0,0461673	0,04940279
malaria	Mauritania	-0,8027585	-0,37155
malaria	Mozambique	0,9838138	1,08385873
malaria	Namibia	-0,7599652	-0,3298758
malaria	Niger	-0,4207408	-0,9854085
malaria	Nigeria	3,30051823	1,77582092
malaria	Rwanda	0,40055629	0,28791859
malaria	Sao Tome and Principe	-0,7799622	-0,3551911
malaria	Senegal	0,1484906	0,51088917
malaria	Sierra Leone	-0,5425775	-0,5598958
malaria	Somalia	-0,550416	-0,1192287
malaria	South Africa	-0,7843359	-0,3365222
malaria	South Sudan	-0,2428738	-0,2605162
malaria	Togo	-0,5490586	-0,3602623
malaria	Uganda	1,54634509	2,90681583
malaria	United Republic of Tanzania	2,21207362	1,93881582
malaria	Zambia	0,62950538	0,17806568
malaria	Zimbabwe	-0,1423438	-0,2739428

Table S4. Z-scores for DAH and averted DALYs for Tuberculosis

Diseases	Country	Total DAH z-score	Aggregate Averted DALYs z-score
tuberculosis	Angola	-0,4537985	0,74859578
tuberculosis	Benin	-0,4819673	-0,2247152
tuberculosis	Botswana	-0,4907975	-0,2309172
tuberculosis	Burkina Faso	-0,2929971	-0,5224847
tuberculosis	Burundi	-0,4366526	-0,0892489
tuberculosis	Cabo Verde	-0,7502456	-0,2368206
tuberculosis	Cameroon	-0,4584746	-0,3398412
tuberculosis	Central African Republic	-0,5165201	-0,4950052
tuberculosis	Chad	-0,6583409	-0,4754275
tuberculosis	Comoros	-0,7468522	-0,2280859
tuberculosis	Congo	-0,6884711	-0,1341013
tuberculosis	Côte d'Ivoire	-0,3670921	-0,1516099
tuberculosis	Democratic Republic of the Congo	1,51656126	0,98712968
tuberculosis	Djibouti	-0,6555144	-0,244045
tuberculosis	Equatorial Guinea	-0,7545413	-0,1697408
tuberculosis	Eritrea	-0,5110757	-0,2007084
tuberculosis	Eswatini	-0,3666521	-0,2512355
tuberculosis	Ethiopia	2,20329161	5,79246331
tuberculosis	Gabon	-0,7444283	-0,2154933
tuberculosis	Gambia	-0,4869119	-0,2574414
tuberculosis	Ghana	0,14369719	-0,2917305
tuberculosis	Guinea	-0,5724027	-0,1835182
tuberculosis	Guinea-Bissau	-0,6098715	-0,2148632
tuberculosis	Kenya	0,97115953	-0,7741042
tuberculosis	Lesotho	-0,3861621	-0,3603206
tuberculosis	Liberia	-0,5167214	-0,1773986
tuberculosis	Madagascar	-0,4829816	-0,2582305
tuberculosis	Malawi	0,73718793	0,16264314
tuberculosis	Mali	-0,5550703	-0,162084
tuberculosis	Mauritania	-0,6695726	-0,2091777
tuberculosis	Mozambique	0,42967324	-0,2803689
tuberculosis	Namibia	-0,1776222	-0,2251763
tuberculosis	Niger	-0,4263084	-0,0946422
tuberculosis	Nigeria	3,4363261	2,24484894
tuberculosis	Rwanda	0,33687366	0,44260268
tuberculosis	Sao Tome and Principe	-0,7337336	-0,2411184
tuberculosis	Senegal	-0,3007086	-0,1659778
tuberculosis	Sierra Leone	-0,4994264	-0,2271433
tuberculosis	Somalia	0,10161183	-1,0526477
tuberculosis	South Africa	3,42053757	-0,1167029
tuberculosis	South Sudan	-0,2681844	-0,0889644
tuberculosis	Togo	-0,6519569	-0,2463069
tuberculosis	Uganda	0,84318813	0,13458468
tuberculosis	United Republic of Tanzania	1,0149453	-0,0290565
tuberculosis	Zambia	0,99415247	0,16159254
tuberculosis	Zimbabwe	0,56285031	-0,8080059

Table S5. Cost-Effectiveness ratio for all Sub-Saharan countries

Country	DAH to averted DALYs (in thousand $)	GDP per capita (x1)	GDP per capita (x3)	Cost effective?
Namibia	-48,82			Dominated
Liberia	-31,38			Dominated
Gambia	-19,85			Dominated
Togo	-18,86			Dominated
South Sudan	-16,55			Dominated
Madagascar	-11,40			Dominated
Eswatini	-8,87			Dominated
Ghana	-4,62			Dominated
Guinea	-2,52			Dominated
Djibouti	-2,50			Dominated
Mozambique	-2,41			Dominated
Lesotho	-2,21			Dominated
Sierra Leone	-1,71			Dominated
Angola	-1,56			Dominated
South Africa	-1,53			Dominated
Chad	-1,52			Dominated
Benin	-1,41			Dominated
Niger	-1,30			Dominated
Somalia	-1,16			Dominated
Cameroon	-0,70			Dominated
Equatorial Guinea	-0,35			Dominated
Nigeria	24,14	4180,89	12542,67	Non Cost-effective
Botswana	11,26	12589,25	37767,74	Very Cost-effective
Sao Tome and Principe	9,95	3295,59	9886,76	Non Cost-effective
Cabo Verde	7,38	5136,53	15409,60	Cost-effective
Mauritania	5,68	4690,69	14072,06	Cost-effective
Eritrea	5,57	N/A	N/A	N/A
Comoros	5,18	2846,49	8539,47	Cost-effective
Mali	4,98	1918,22	5754,66	Cost-effective
Zambia	3,42	2661,99	7985,96	Cost-effective
Rwanda	3,33	1320,27	3960,82	Cost-effective
Guinea-Bissau	3,29	1805,04	5415,12	Cost-effective
Kenya	3,20	3569,45	10708,36	Very Cost-effective
Central African Republic	2,63	1039,82	3119,45	Cost-effective
Gabon	2,07	16015,05	48045,16	Very Cost-effective
Senegal	1,66	2737,54	8212,61	Very Cost-effective
Zimbabwe	1,57	2291,81	6875,44	Very Cost-effective
United Republic of Tanzania	1,46	1838,03	5514,10	Very Cost-effective
Ethiopia	1,41	1141,83	3425,50	Cost-effective
Burkina Faso	1,37	1584,38	4753,15	Very Cost-effective
Malawi	1,22	1245,09	3735,28	Very Cost-effective
Côte d'Ivoire	1,16	3974,56	11923,68	Very Cost-effective
Uganda	0,99	1635,31	4905,94	Very Cost-effective

Democratic Republic of the Congo	0,64	890,45	2671,34	Very Cost-effective
Congo	0,53	4673,86	14021,59	Very Cost-effective
Burundi	0,50	835,57	2506,70	Very Cost-effective
